# 4-Phenylbutyric acid extends the gold time of uncontrolled hemorrhagic shock at high altitude by alleviating vital organ injury

**DOI:** 10.1186/s40635-025-00833-w

**Published:** 2025-12-19

**Authors:** Jie Zhang, Xiao-Yong Peng, Yue Wu, Qing-Hui Li, Xin-Ming Xiang, Yuan-Qun Zhou, Yu Zhu, Zi-Sen Zhang, Hao-Yue Deng, Li Wang, Liang-Ming Liu, Tao Li

**Affiliations:** Shock and Transfusion Department of Research Institute of Surgery, Daping Hospital, Army Medical University (Third Military Medical University), Chongqing, 400042 People’s Republic of China

**Keywords:** 4-Phenylbutyric acid, Uncontrolled hemorrhagic shock, Gold time, High altitude

## Abstract

**Background:**

Uncontrolled hemorrhagic shock (UHS) is prevalent in military operations, disaster relief, and traffic accidents at high altitudes. Due to reduced tolerance to resuscitation fluids and prolonged evacuation times, its management poses substantial challenges. Whether 4-phenylbutyric acid (PBA) can protect vital organ function and extend the golden period for UHS at high altitudes remains unclear.

**Methods:**

Rats airlifted from Chongqing to Lhasa were used to establish a UHS model. The experiment consisted of three parts: Part 1 investigated PBA’s effect on extending the golden period (prehospital treatment window). Specifically, using a high-altitude rat model of UHS, we observe the duration that PBA + LR maintains mean arterial pressure (MAP) at 50–60 mmHg without definitive hemostasis; Parts 2 and 3 involve hypotensive maintenance of MAP at 50–60 mmHg for 1 and 2 h, respectively, prior to definitive hemostasis, simulating 1-h and 2-h prehospital phases. After hypotensive maintenance, definitive hemostasis is performed. Parameters including vital organ injury markers, blood gas profiles, and survival rates were assessed.

**Results:**

In Part 1, PBA (20 mg/kg) reduced blood loss by 13.3% (from 53.6 ± 2.4% to 45.28 ± 3.4%) and resuscitation fluid volume by 28% compared to LR alone. PBA (20 mg/kg) prolonged the duration of sustained hypotensive resuscitation by 243% (from 39 ± 4.6 min to 134 ± 10.6 min) compared to LR, stabilized hemodynamics, and improved 2-h survival from 12.5% to 62.5%. In Part 2, 20 mg/kg PBA attenuated vital organ damage, increased 72-h survival from 18.7% (LR group) to 50% (20 mg/kg PBA group), and meanwhile reduced blood loss by 7.7% and resuscitation fluid volume by 16.3% compared to LR alone. In Part 3, despite extending hypotensive resuscitation to 2 h, PBA still significantly ameliorated organ function, reduced blood loss, decreased fluid administration, and enhanced 72-h survival in rats from 0% (LR group) to 31.25% (20 mg/kg PBA group).

**Conclusion:**

PBA administration during hypotensive resuscitation protects vital organs (heart, liver, kidney), reduces pulmonary and cerebral edema incidence, and significantly extends the golden period for UHS at high altitudes.

**Supplementary Information:**

The online version contains supplementary material available at 10.1186/s40635-025-00833-w.

## Introduction

Worldwide, research on the management of uncontrolled hemorrhagic shock (UHS) in high-altitude environments remains limited. Although, significant advances have been made in the treatment of UHS in low-altitude (plain) regions including the proposal of damage control resuscitation and the establishment and refinement of fluid resuscitation principles and techniques [1], these techniques for UHS management in conventional environments cannot be completely applied to the treatment of high-altitude uncontrolled hemorrhagic shock (HAUHS) [2, 3]. This is attributable to the unique pathophysiological characteristics of HAUHS, such as decreased tolerance to resuscitation fluids and increased susceptibility to pulmonary edema and cerebral edema [4, 5], which further lead to multiple organ dysfunction. Undoubtedly, however, whether in high-altitude or low-altitude areas, fluid resuscitation remains the cornerstone of UHS treatment. Therefore, the key to addressing the treatment of HAUHS lies in how to combine drug administration in the early stage of shock on the basis of conventional resuscitation fluids to protect organ function and extend the 1-h golden period (i.e., the critical prehospital treatment window) in patients with HAUHS [6, 7].

Hypoxia is a hallmark feature of high-altitude environments, and existing literature has confirmed that it triggers stress responses such as endoplasmic reticulum stress and oxidative stress [8], and these processes we previously linked to UHS-induced organ injury. 4-phenylbutyrate (PBA), a compound well-recognized for its ability to inhibit endoplasmic reticulum stress (ERS) and oxidative stress [9], is FDA-approved for the treatment of urea cycle disorders at a recommended dose range of 9.9 to 13 g/m^2^ [10]. Recently, extensive biological activities of PBA have been identified, including anti-inflammatory effects [11], and modulation of cellular lipid metabolism [12]. Additionally, our prior studies demonstrated that PBA protects against vital organ dysfunction induced by hypoxia and inflammation through inhibiting endoplasmic reticulum stress and oxidative stress [13, 14]. However, whether PBA can alleviate vital organ injury following HAUHS and extend the golden period (i.e., the prehospital treatment window) remains unclear. To explore the potential beneficial effects of PBA in this context, an UHS model was established in rats at Lhasa (3600 m altitude), and the protective role of PBA in HSUHA was evaluated.

## Materials and methods

### Ethical approval of the study

The present study conformed to the principles of the “Guide for the Care and Use of Laboratory Animals” (Eighth Edition, 2011, National Academies Press, Washington DC) and was approved by the Research Council and Animal Care and Use Committee of the Research Institute of Surgery [Daping Hospital, Third Military Medical University (Army Medical University), Chongqing, P.R. China] (AMUWEC20226303). The ARRIVE guidelines' recommendations for research were followed [15].

### Animal management

A total of 224 male and female Sprague–Dawley rats (220–240 g) were purchased from the Animal Center of Research Institute of Surgery, Third Military Medical University (Army Medical University) and utilized in the present study. All rats were airlifted from Chongqing to Lhasa in a specially designed cage, with free access to food and water. After arriving in Lhasa, all the rats were acclimatized to the plateau environment for 48 h in a room at constant humidity (60 ± 5%), temperature (24 ± 1 °C), and light cycle (6 am–6 pm) and fed standard pellet diets ad libitum.

### Uncontrolled hemorrhagic shock rat model

Rats were anaesthetized with sodium pentobarbital (initial dose, 30 mg/kg, intraperitoneally), with additional doses administered to maintain areflexia to needle stimulation. Aseptic techniques were adopted for all surgical procedures. Polyethylene catheters (outer diameter 0.965 mm, inner diameter 0.58 mm) were inserted into the right femoral artery (for mean arterial pressure [MAP] monitoring) and vein (for drug administration). Intravenous heparin (1000 U/kg) was given to prevent thrombosis. A midline laparotomy exposed the spleen. UHS was induced as previously described [16]: cross-transection of splenic parenchyma between the two major branches, of the splenic artery, concurrent transection of one main branch, and unimpeded intra-abdominal bleeding. Shock was established once MAP declined to 40 mmHg, initiating subsequent experiments.

### Experimental protocols

#### Part 1: the effects PBA on prehospital management of high-altitude uncontrolled hemorrhagic shock (HAUHS)

**Fig. 1:**
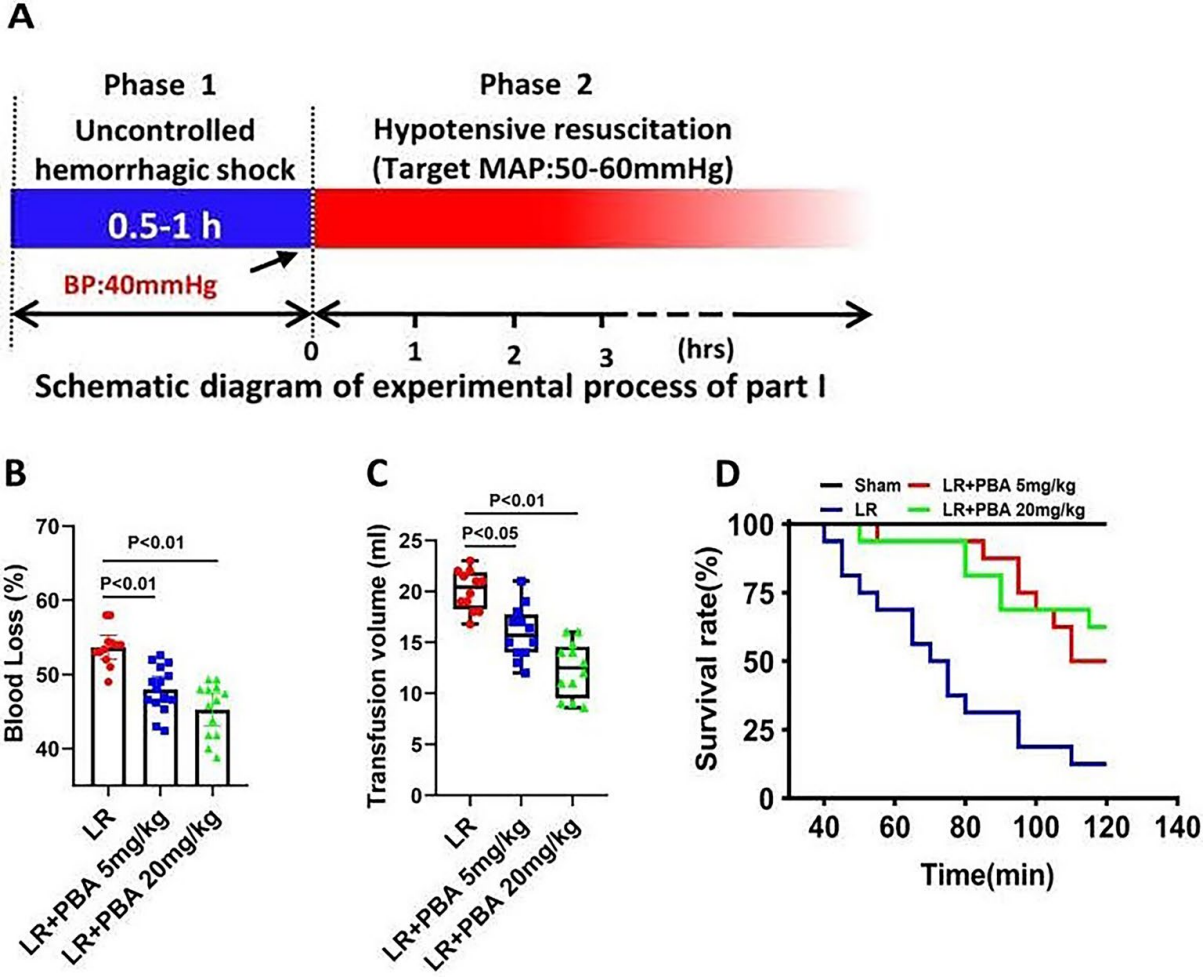
The effect of PBA on uncontrolled hemorrhagic shock rats at high altitude. **A** Schematic diagram of the experimental process of Part 1; **B**, C the blood loss (**B**) (data are means ± SD (*n* ≥ 12 rats/group)), and transfusion volume of LR (**C**) (data are median with interquartile range (*n* ≥ 12 rats/group)). **D** The survival rate of uncontrolled hemorrhagic shock rats after 2-h hypotensive resuscitation (*n* = 16). **p < 0.01 vs LR groups. *p* < 0.05 was considered significant. S: uncontrolled hemorrhagic shock; LR: lactate Ringer’s solution; NT: non-treatment

Forty-eight Sprague–Dawley rats were randomly divided into three groups: lactate Ringer’s solution (LR) resuscitation groups, LR resuscitation combined with 5 mg/kg PBA groups and LR resuscitation combined with 20 mg/kg PBA groups. Rats in all groups underwent the management process as shown in Fig. 1A. Briefly, in Phase 1, an UHS model (a MAP of 40 mmHg) was created within one hour by cutting the spleen. At the start of Phase 2, lactated LR, LR combined with 5 mg/kg PBA, or LR combined with 20 mg/kg PBA were administered to maintain a mean arterial pressure (MAP) of 50–60 mmHg. In cases where maintenance of a MAP of 50–60 mmHg failed, the following parameters were recorded for each rat: blood loss volume, the volume of resuscitation fluid, and the duration of MAP maintenance at 50–60 mmHg.

#### Part 2: the effects of PBA on vital organ function in rats with HAUHS

**Fig. 2:**
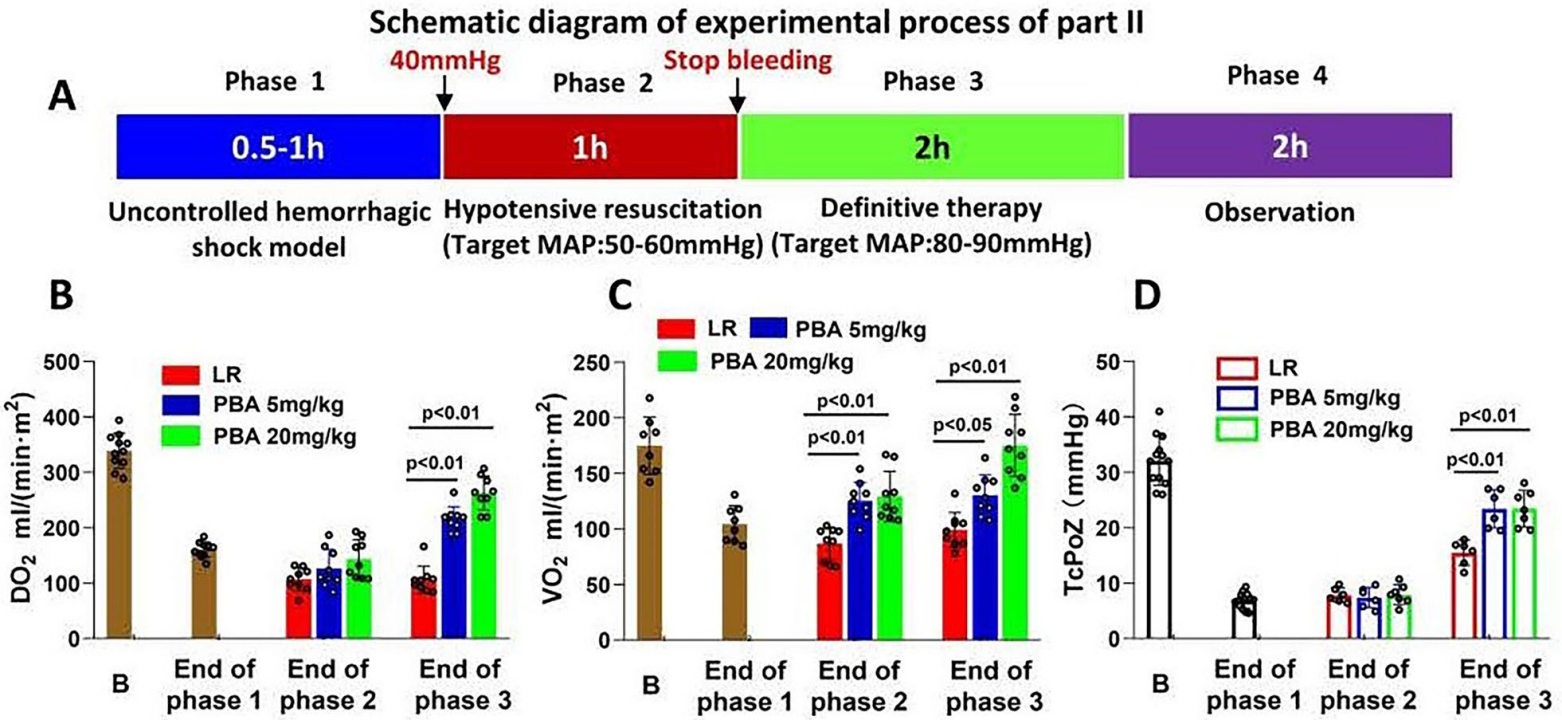
The effect of PBA on blood gas and oxygen delivery and consumption of uncontrolled hemorrhagic shock rats underwent 1-h hypotensive resuscitation. **A** Schematic diagram of the experimental process of Part 2. **B**–**D** Oxygen delivery (DO2) (**B**), oxygen consumption (VO2) (**C**) and the transcutaneous partial pressure of oxygen (PtcO2) (**D**) of rats at the end of each phase. Data are means ± SD (*n* ≥ 8 rats/group). *p* < 0.05 was considered significant. B: baseline; LR: lactate Ringer’s solution

In this section, 48 Sprague–Dawley rats, randomly divided into three groups (LR, LR + 5 mg/kg PBA, LR + 20 mg/kg PBA), underwent the experimental process as shown in Fig. 2A. Phase 1 involved creating the UHS model. During Phase 2, lactated LR, LR combined with 5 or 20 mg/kg PBA was administered to maintain MAP at 50–60 mmHg for 1 h. At the end of Phase 2, the volume of blood loss and resuscitation fluid was recorded. Phase 3 began with stopping bleeding by ligating the proximal cardiac end of the splenic artery, followed by lactated LR treatment for two hours. At the end of Phase 3, the parameters of cardiac injury, kidney injury and liver function were measured, and the pulmonary and cerebral edema were evaluated. At the end of Phase 4, the 72-h survival rate was recorded. The cardiac output, transcutaneous oxygen pressure (TcPoZ), blood gas and the liver and kidney blood flow were measured at the end of each phase.

#### Part 3: the effect of PBA on extending the golden period (prehospital treatment phase) in rats with HAUHS

In accordance with the experimental flowchart in Part 2 (Fig. 2A), only the duration of hypotensive resuscitation was extended to 2 h as shown in Fig. 5A. A total of 48 rats were randomly divided into three groups (LR, LR + 5 mg/kg PBA, LR + 20 mg/kg PBA). At the end of Phase 2, blood loss volume and the volume of resuscitation fluid was recorded. At the end of Phase 3, the parameters of cardiac injury, kidney injury and liver function were measured, and the pulmonary and cerebral edema were evaluated. At the end of Phase 4, the 72-h survival rate was recorded.

### The measurement of parameters

Mean arterial pressure (MAP): the MAP was measured every 5 min by mercury sphygmomanometer via polyethylene catheters which were connected into the right femoral artery. Transcutaneous Oxygen Pressure (TcPoZ): the abdominal hair was removed and transcutaneous oxygen pressure was measured by The PeriFlux 5000 system (with a tcpO_2_ unit, PF5040, PERIMED Co., Ltd., Sweden) under an air-insulated condition. Cardiac Output: the cardiac output (CO) was measured with thermodilution techniques. Briefly, a thermodilution probe was inserted into the aorta ascendens of the rat, and 0.3 ml ice-bathed saline was injected through the right external jugular vein catheter. The CO was determined using a CO analyzer (Power Laboratory, AD Instruments, Australia). Oxygen delivery (DO_2_) and consumption (VO_2_) were calculated with the following equations: DO_2_ = CI × 13.4 × [Hb] × SaO_2_; VO_2_ = CI × 13.4 × [Hb] × (SaO_2_-SvO_2_). The Liver and Kidney Blood Flow: the rats underwent a laparotomy and the blood flow in the liver and kidney was measured by a laser speckle technique (PeriScan PIM3 System; Stockholm, Sweden). Blood Gases: at every time point, 1 ml of arterial blood was obtained by a heparinized and hermetic injector via femoral artery cannula. A blood gas analyzer (ABL90FLEX, Radiometer, Denmark) was used to measure the PaO_2_, SaO_2_, PCO_2_, PH, HCO_3_^−^, bases excess (BE), and lactic acid in the blood. Vital Organ Function: 3 ml of arterial blood was obtained via femoral artery cannula, which was then subjected to centrifugation at 3500 revolutions per minute (rpm) at room temperature. The serum supernatant in the upper level was analyzed by a biochemical analyzer (DX800, Beckman Coulter, 250 S. Kraemer Boulevard Brea, CA, United States). The damage degree of the liver was reflected by the level of glutamic-pyruvic transaminase (ALT) and glutamic-oxalacetic transaminase (AST) in blood. The kidney injury was reflected by urea and creatinine (CREA) and the cardiac injury was reflected by troponin T (TNT) in blood. Pulmonary and Cerebral Edema: the water content of the brain and lung was determined by the formula: water content = (wet weight − dry weight)/wet weight.

### Hematoxylin–eosin (HE) staining for rat lung tissues

After the rats were euthanized, lung tissues were excised immediately, trimmed to 3–5 mm thickness, and fixed in 4% paraformaldehyde in PBS (pH7.4) at 4 °C for 24 h. Post-fixation, tissues were dehydrated at RT with a graded ethanol series: 70% (15 min), 80% (15 min), 90% (15 min), 95% (2 × 15 min), 100% (2 × 20 min). Dehydrated tissues were cleared in xylene (2 × 20 min) and embedded in paraffin (56–58 °C). Serial 5-μm sections were cut with a microtome, mounted on poly-L-lysine-coated slides, and baked at 60 °C for 1 h to enhance adhesion. For staining: sections were dewaxed in xylene (2 × 10 min), rehydrated via reversed ethanol gradient, then rinsed with distilled water (2 × 5 min). Stain with Harris hematoxylin for 8 min, differentiate in 1% hydrochloric acid–ethanol (v/v) for 10 s, rinse with tap water for 5 min, and blue with 0.1% ammonia water for 30 s, then rinsed with distilled water (2 × 3 min). Subsequently, stain with 0.5% eosin Y for 3 min, dehydrate in 95% ethanol (2 × 5 min) and 100% ethanol (2 × 5 min), clear in xylene (2 × 10 min). Finally, mount with neutral balsam and observe under a light microscope (Leica SP5, Germany) for morphological assessment.

### Statistical analysis

The blood loss, MAP, DO_2_, VO_2_, TcPoZ, CO, parameters of organ function, blood gas parameters, blood flow, survival time were presented as the means ± standard deviations (M ± SD) of n observations. Transfusion volume was presented with the median and interquartile range. The statistical differences among groups were analyzed using two-factor variance analysis, followed by the post hoc Tukey test (SPSS v15, SPSS Inc, Chicago, IL, United States) for multiple comparisons between two groups. Survival rate was analyzed by Kaplan–Meier survival analysis and the log-rank test. All data underwent the Kolmogorov–Smirnov normality test and the Bartlett homoscedasticity test. *p* < 0.05 and *p* < 0.01 were considered significant.

## Results

### Part 1: PBA exerts beneficial effects in prehospital management of high-altitude uncontrolled hemorrhagic shock (HAUHS)

Results showed that PBA administration during the hypotensive resuscitation phase (Phase 2) significantly reduced blood loss in rats with UHS (LR group vs. 20 mg/kg PBA group: 53.6 ± 2.4% vs. 45.28 ± 3.4%, *p* < 0.01) (Fig. 1B). Median and interquartile analysis revealed a decrease in resuscitation fluid volume required to maintain target MAP (50–60 mmHg) during Phase 2 following PBA treatment (LR group vs. 20 mg/kg PBA group: 20.5 ml (17.9 ml, 22.1 ml) vs, 13.6 ml (9.2 ml, 14.6 ml)) (Fig. 1C). Additionally, mean ± standard deviation (SD) analysis demonstrated that PBA stabilized blood pressure in rats with UHS during hypotensive resuscitation, with a significant prolongation of the stable maintenance period (Fig S1A). PBA administration during Phase 2 also significantly improved both 1-h and 2-h survival rates (LR group vs. 20 mg/kg PBA group: 68.75% vs. 93.75%, *p* < 0.01) in the UHS model (Fig. 1D). These findings indicate that PBA application during hypotensive resuscitation stabilizes hemodynamics, reduces blood loss and resuscitation fluid requirements, and improves 2-h survival in rats with HAUHS.

### Part 2: PBA exerts favorable effects on vital organ function in rats with HAUHS

First, the effects of PBA on global oxygen delivery (DO_2_), global oxygen consumption (VO_2_), transcutaneous oxygen pressure (TcPO_2_), and arterial blood gas (ABG) parameters were assessed. Results showed that compared with LR resuscitation alone, PBA treatment significantly enhanced DO_2_ in UHS rats at the end of definitive therapy (LR group vs. 20 mg/kg PBA group: 101.5 ± 28.5 vs. 278.5 ± 31.2, *p* < 0.01) (Fig. 2B). Meanwhile, PBA gradually elevated VO_2_ by the end of hypotensive resuscitation (Phase 2), with this effect persisting until the end of Phase 3(LR group vs. 20 mg/kg PBA group: 102.3 ± 19.7 vs. 176.6 ± 35.6, *p* < 0.01) (Fig. 2C). TcPO_2_ was increased at the end of Phase 3 following PBA administration (LR group vs. 20 mg/kg PBA group: 15.6 ± 2.1 vs. 23.9 ± 4.3, *p* < 0.01) (Fig. 2D). For ABG parameters, both PaO_2_ and SaO_2_ were significantly higher at the end of hypotensive resuscitation (Phase 2) with PBA treatment (PaO_2_:LR group vs. 20 mg/kg PBA group: 89.4 ± 5.3 vs. 94.4.9 ± 3.8, *p* < 0.01; SaO_2_:LR group vs. 20 mg/kg PBA group: 88.3 ± 2.3 vs. 95.1 ± 2.9, *p* < 0.01). With continued PBA administration during definitive therapy (Phase 3), PaO_2_ and SaO_2_ were further restored by the end of Phase 3 (Table 1). Additionally, PBA reduced blood levels of HCO_3_⁻ (LR group vs. 20 mg/kg PBA group: 13.4 ± 3.5 vs. 16.5 ± 3.3, *p* < 0.05) and cLac (LR group vs. 20 mg/kg PBA group: 13.5 ± 1.6 vs. 11.9 ± 1.3, *p* < 0.05) (Table 1).

**Table 1:** The changes of blood gases (*n* = 8). #: p<0.05, ##: P<0.01 vs. CT group

Groups	Baseline	End of Phase I	End of Phase II	End of Phase III
PaO_2_ (mmHg)				
CT	98.7 ± 3.2	81.3 ± 4.6	84.6 ± 3.6	89.4 ± 5.3
PBA 5 mg/kg			86.3 ± 3.1#	93.2 ± 4.6##
PBA 20 mg/kg			85.3 ± 2.7#	94.4 ± 3.8##
SaO_2_ (%)				
CT	96.4 ± 3.1	79.2 ± 1.9	80.1 ± 2.7	88.3 ± 2.3
PBA 5 mg/kg			84.6 ± 4.1##	93.2 ± 3.8##
PBA 20 mg/kg			84.6 ± 3.2##	95.1 ± 2.9##
PCO_2_ (mmHg)				
CT	30.43 ± 3.1	29.4 ± 2.1	31.4 ± 3.2	32.3 ± 4.3
PBA 5 mg/kg			32.1 ± 3.7	33.2 ± 2.8
PBA 20 mg/kg			31.9 ± 2.4	31.7 ± 4.3
PH				
CT	7.34 ± 0.04	7.33 ± 0.03	7.32 ± 0.04	7.29 ± 0.05
PBA 5 mg/kg			7.32 ± 0.06	7.28 ± 0.04
PBA 20 mg/kg			7.31 ± 0.03	7.30 ± 0.03
HCO_3_^−^ (mmol/L)				
CT	17.3 ± 3.5	16.3 ± 2.9	14.9 ± 3.2	13.4 ± 3.5
PBA 5 mg/kg			15.3 ± 4.2	16.7 ± 3.1#
PBA 20 mg/kg			15.6 ± 2.5#	16.5 ± 3.3#
BE (mmol/L)				
CT	− 1.8 ± 0.9	− 1.9 ± 0.8	− 2.0 ± 0.7	− 2.3 ± 1.1
PBA 5 mg/kg			− 2.1 ± 1.1	− 2.2 ± 1.2
PBA 20 mg/kg			− 2.1 ± 0.9	− 2.3 ± 0.9
cLac (mmol/L)				
CT	11.4 ± 1.7	11.1 ± 1.5	11.6 ± 2.1	13.5 ± 1.6
PBA 5 mg/kg			12.3 ± 1.9	11.7 ± 0.9##
PBA 20 mg/kg			11.9 ± 1.7	11.9 ± 1.3#

**Fig. 3:**
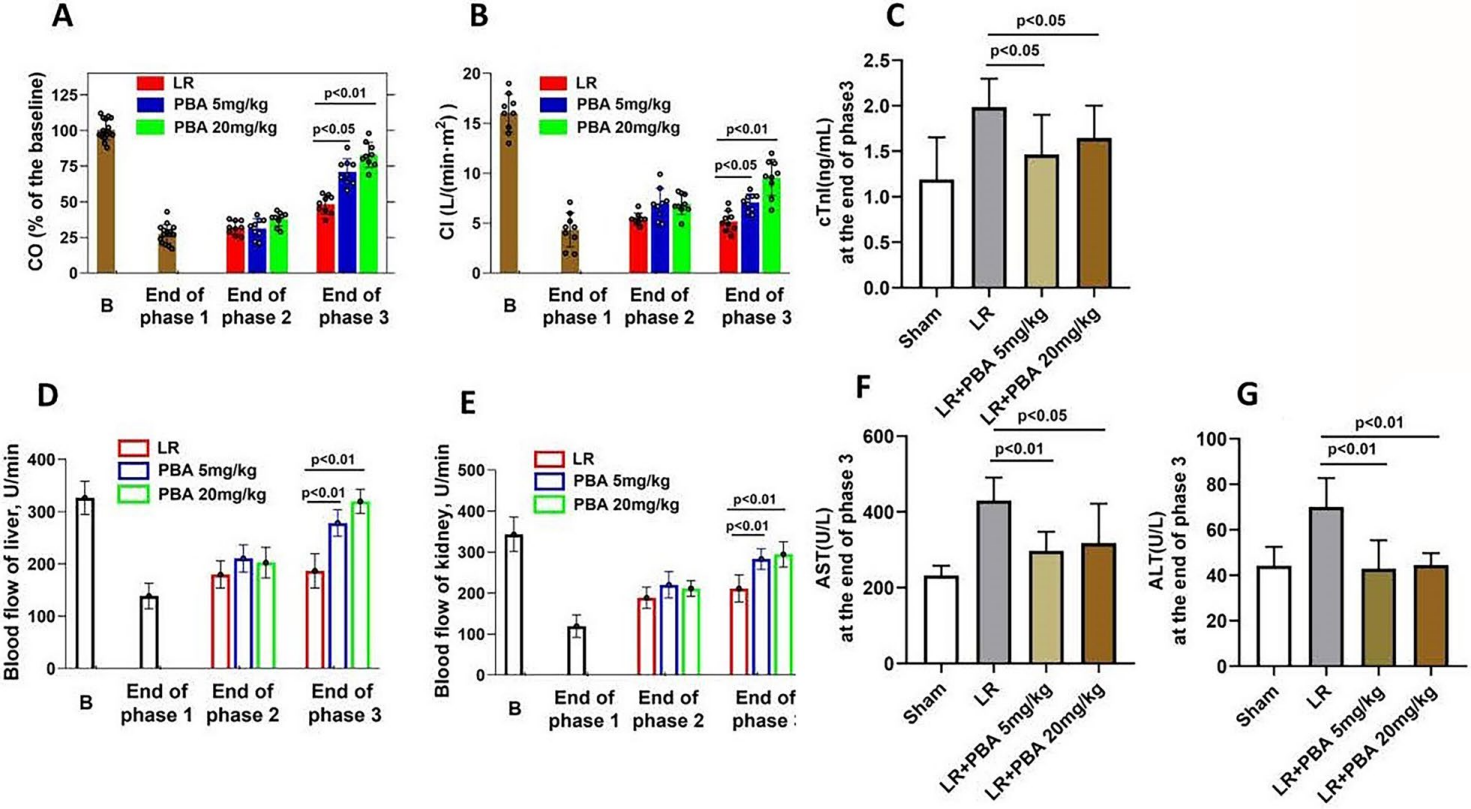
The effect of PBA on vital organ function of uncontrolled hemorrhagic shock rats underwent 1-h hypotensive resuscitation. **A**, **B** The CO (**A**) and CI (**B**) of rats at the end of each phase; **C** cardiac injury index (Tni) of rats at the end of Phase III; **D** the liver blood flow of hemorrhagic shock rats. **E**, **F** the liver injury index (AST) and (ALT); **G** the renal blood flow of hemorrhagic shock rats; **H**, **I** the renal function. Data are means ± SD (*n* ≥ 8 rats/group). *p* < 0.05 was considered significant. B: baseline; LR: lactate Ringer’s solution

Second, the impact of PBA on vital organ function was evaluated. UHS reduced cardiac output (CO) and cardiac index (CI) in rats. PBA administration during hypotensive resuscitation significantly increased CO (LR group vs. 20 mg/kg PBA group: 47.2 ± 8.6 vs. 82.2 ± 10.2, *p* < 0.01) and CI by the end of Phase 3, whereas LR alone only partially rescued CO by this time point (Fig. 3A, B). The cardiac injury marker cTnI was markedly decreased with PBA treatment (LR group vs. 20 mg/kg PBA group: 1.96 ± 0.31 vs. 1.58 ± 0.29, *p* < 0.05) (Fig. 3C). Liver and kidney blood flow declined sharply by the end of Phase 1. Hypotensive resuscitation with LR alone or LR combined with PBA partially restored liver and kidney blood flow by the end of Phase 2. However, by the end of Phase 3, PBA exerted a significantly greater effect on restoring liver and kidney blood flow (LR group vs. 20 mg/kg PBA group: 208.4 ± 28.1 vs. 298.3 ± 26.5, *p* < 0.01), whereas LR alone failed to further enhance these parameters (Fig. 3D, E). Concomitantly, PBA reduced blood levels of the liver injury markers AST and ALT (Fig. 3F, G), though it had no significant impact on renal function indices (Figure not shown).

**Fig. 4:**
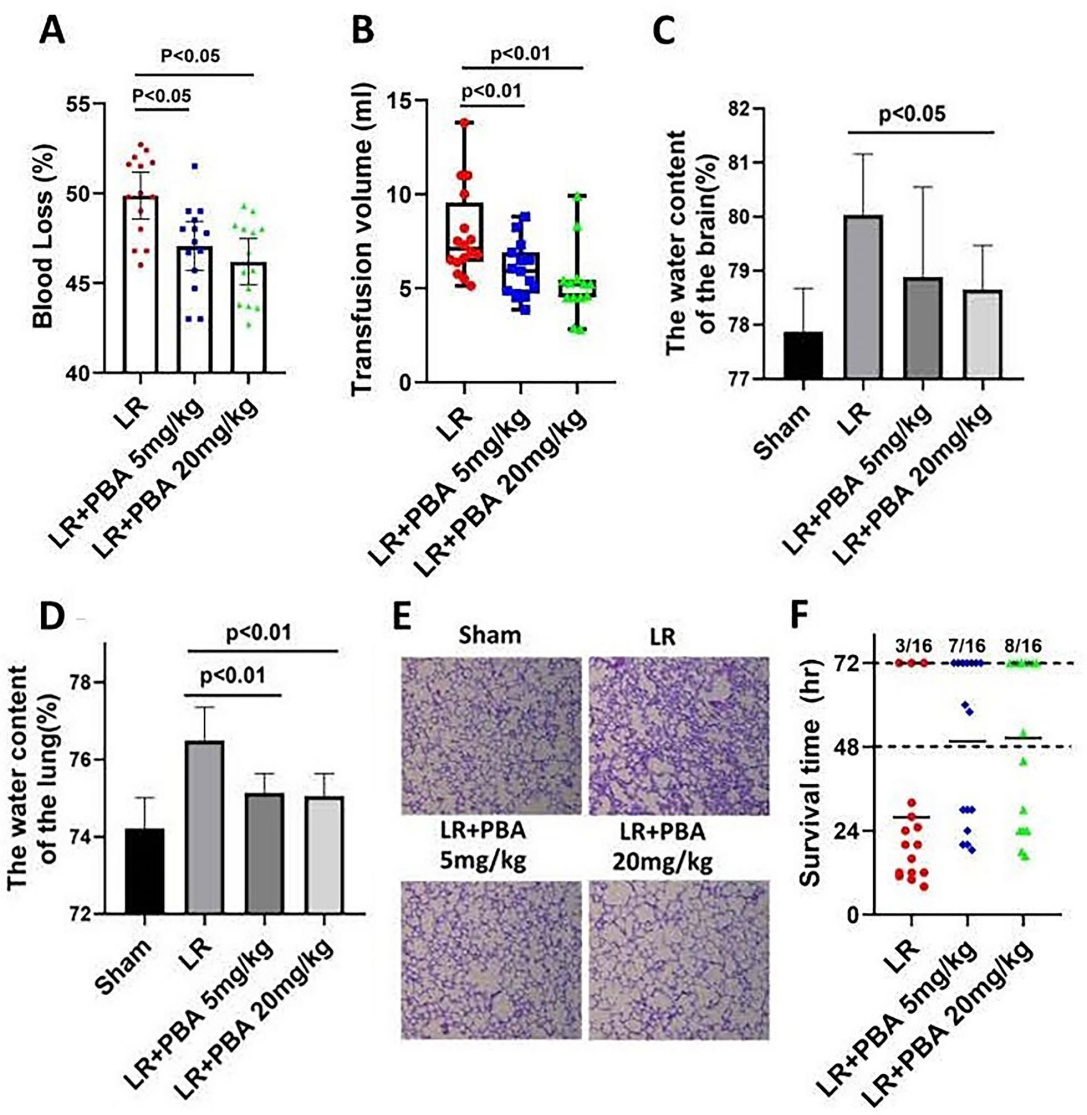
The effect of PBA on the rats that underwent 1-h hypotensive resuscitation. **A**, **B** The blood loss (**A**) (data are means ± SD), transfusion volume of LR (B) (data are median with interquartile range), **C**, **D** water content of lung and brain. **E** Inflammatory cell infiltration in lung tissue. **F** 72-h survival rate. Data are means ± SD (*n* ≥ 8 rats/group). *p* < 0.05 was considered significant. S: uncontrolled hemorrhagic shock; LR: lactate Ringer’s solution

Finally, we analyzed the total blood loss and resuscitation fluid volume throughout the experiment, examined the effects of PBA on pulmonary and cerebral edema, and recorded the survival time. PBA administration reduced blood loss, with a more pronounced effect at higher doses (blood loss (%): LR group vs. 20 mg/kg PBA group: 49.8% ± 1.4 vs. 45.9% ± 1.6, *p* < 0.05) (Fig. 4A). Mean ± standard deviation (SD) analysis revealed a reduction in total resuscitation fluid volume with PBA treatment (Fig. 4B). Lung and brain water content were increased in UHS rats resuscitated with LR alone, whereas PBA administration significantly reduced these parameters (Fig. 4C, D). PBA markedly decreased pulmonary inflammatory cell infiltration (Fig. 4E). Moreover, PBA administration during Phases 2 significantly improved the 72-h survival rate (LR group vs. 20 mg/kg PBA group: 18.75% vs. 50%) of rats with HAUHS (Fig. 4F).

### Part 3: early PBA administration extends the golden period (prehospital treatment phase) to 2 h in rats with HAUHS

**Fig. 5:**
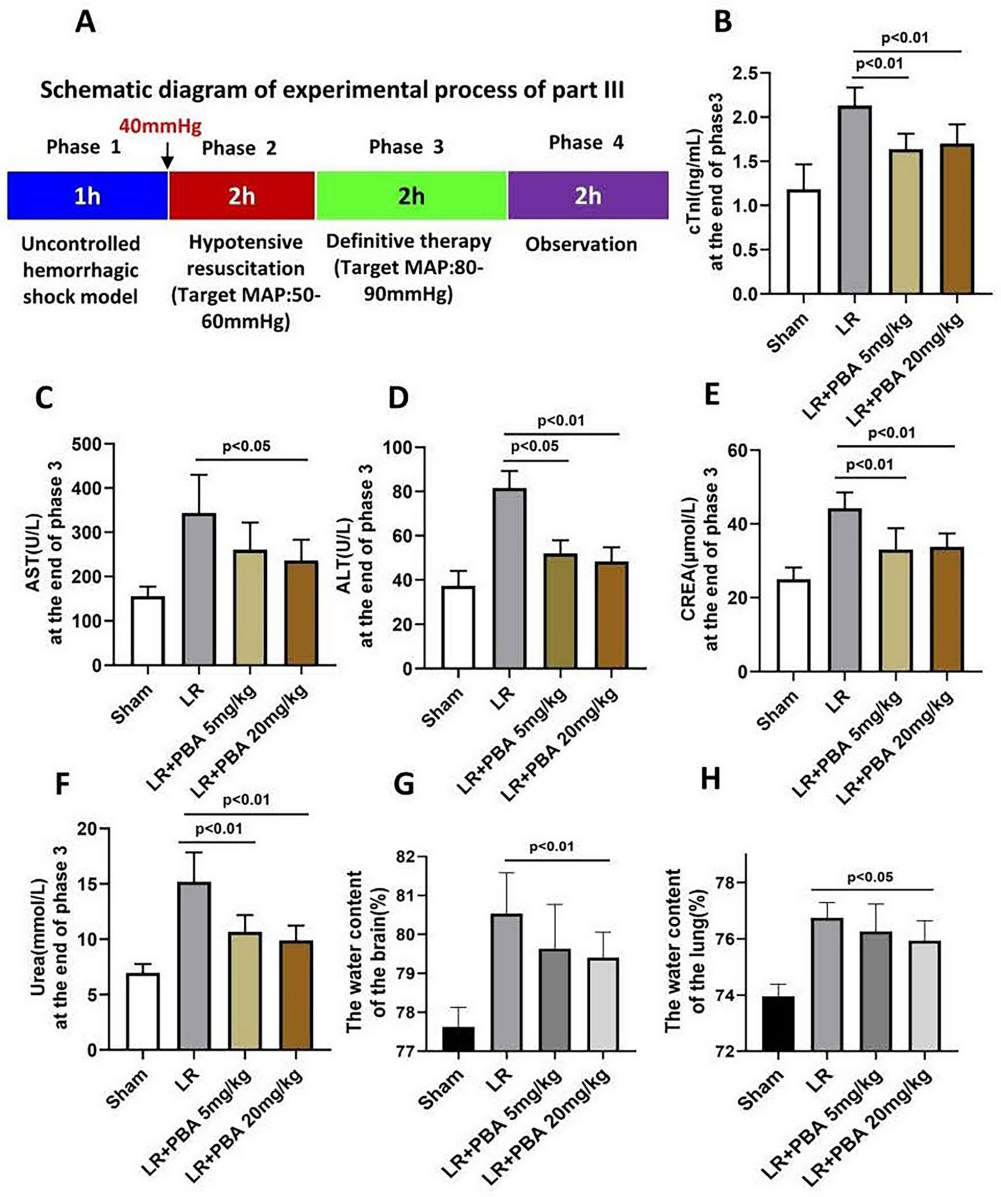
The effect of PBA on vital organ function of uncontrolled hemorrhagic shock rats undergoing 2-h hypotensive resuscitation. **A** Schematic diagram of the experimental process of Part 3; **B** cardiac injury index (TnI) of rats at the end of Phase III; **C**, **D** the liver injury index (AST) and (ALT) at the end of Phase III; **E**, **F** the renal function at the end of Phase III; **G**, **H** water content of the lung and brain. Data are means ± SD (*n* = 8 rats/group). *p* < 0.05 was considered significant

Based on the above findings, we extended the hypotensive resuscitation phase to 2 h to mimic the prolonged prehospital treatment period resulting from harsh environments and limited evacuation capabilities in high-altitude disaster relief or military operations. The experimental protocol is outlined in Fig. 5A. Results showed that vital organ injury was more severe in rats subjected to 2-h hypotensive resuscitation compared to those with 1-h hypotensive resuscitation (Fig. 5 vs. Fig. 3). However, compared with LR resuscitation alone, PBA treatment alleviated cardiac injury (Fig. 5B) and liver injury (Fig. 5C, D), and preserved renal function (Fig. 5E, F). Additionally, PBA reduced lung and brain water content, mitigating pulmonary and cerebral edema (Fig. 5G, H).

**Fig. 6:**
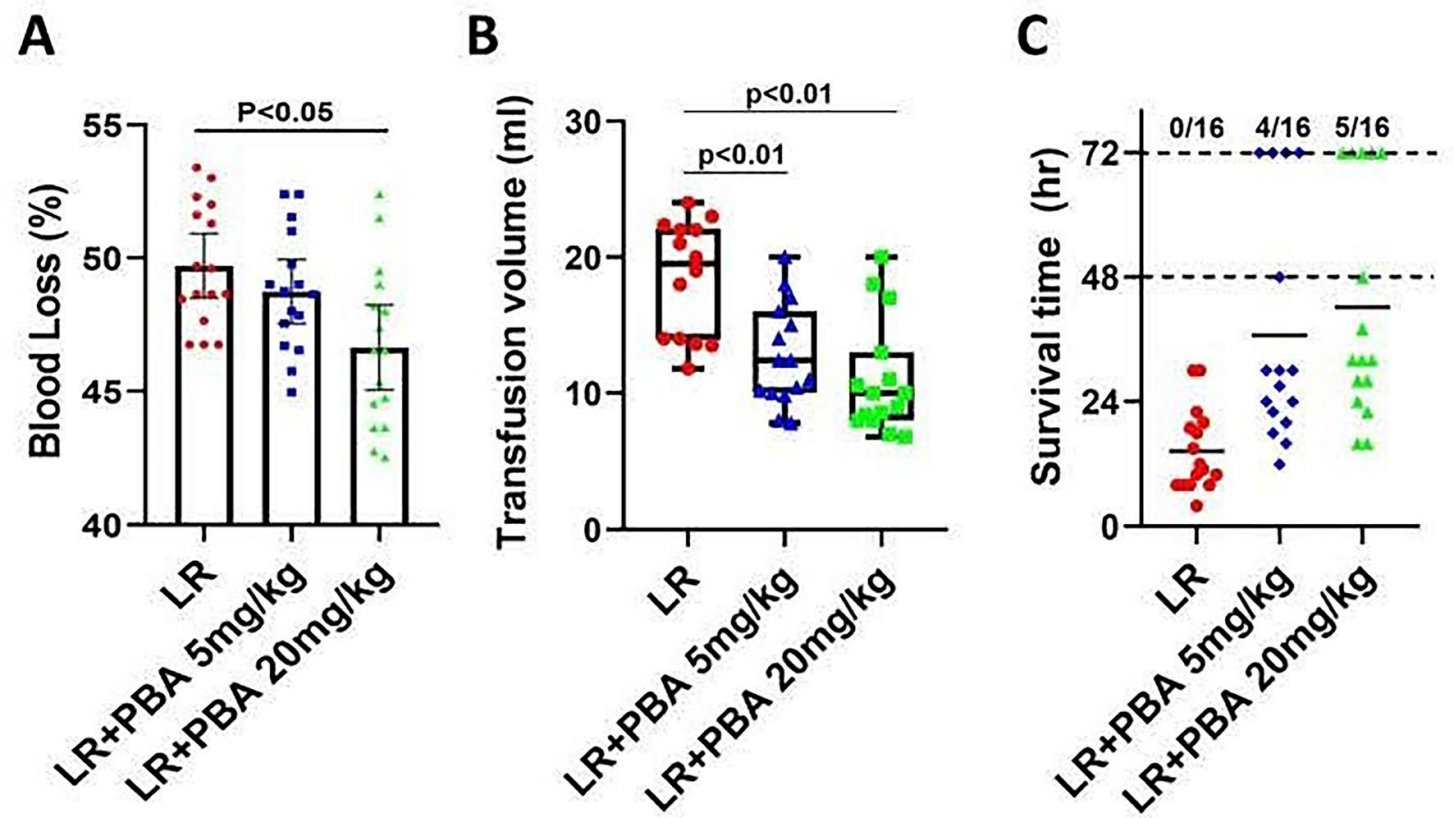
The effect of PBA on the 72-h survival rate of rats that underwent 2-h hypotensive resuscitation. **A**, **B** The blood loss (**A**) (data are means ± SD), transfusion volume of LR (**B**) (data are median with interquartile range). **C** 72-h survival rate. S: uncontrolled hemorrhagic shock; LR: lactate Ringer’s solution

PBA treatment decreased blood loss during the resuscitation phase (Fig. 6A). Mean ± standard deviation (SD) analysis revealed a reduction in total resuscitation fluid volume with PBA co-administration (Fig. 6B). Furthermore, LR resuscitation combined with PBA significantly improved the 72-h survival rate (LR group vs. 20 mg/kg PBA group: 0% vs. 31.25%), whereas LR alone failed to sustain survival beyond 48 h in any of the 16 rats (Fig. 6C).

Finally, we used a bioinformatics approach to retrieve 3375 shock-related disease genes from the GeneCards and DisGeNET databases, and 136 potential targets of 4-phenylbutyric acid (PBA) from the SEA and Swiss Target databases. Venn analysis identified 61 overlapping targets (Fig. S1B). Gene Ontology (GO) functional enrichment analysis further supported our experimental results, indicating that PBA may exert effects via anti-inflammatory activity in this study (Fig. S1C). To validate this hypothesis, we established a rat model of HAUHS via a hypobaric oxygen chamber (5000 m simulation, 48 h) and measured PBA’s impact on plasma interleukin-6 (IL-6) and tumor necrosis factor-α (TNF-α) concentrations. Specifically, results confirmed that plasma IL-6 and TNF-α levels increased significantly following shock. Treatment with LR solution alone failed to reduce IL-6 and TNF-α levels, whereas PBA treatment significantly decreased the plasma concentrations of these two cytokines (Fig. S1D, E). This further confirmed that the mechanism underlying PBA-mediated organ protection may be associated with inflammation inhibition.

## Discussion

Prehospital management of uncontrolled hemorrhagic shock (UHS) in resource-limited settings, such as high-altitude military operations, remote disaster zones, or regions with constrained medical infrastructure, presents unique challenges, primarily stemming from delayed access to blood products, limited resuscitation resources, and exacerbated pathophysiology due to hypoxia and environmental stress [17, 18]. In such contexts, the ability to stabilize hemodynamics, minimize organ injury, and extend the "golden period" for definitive care becomes pivotal. Our findings suggest 4-phenylbutyric acid (PBA) may serve as a valuable adjunct to crystalloid resuscitation in addressing these challenges.

PBA is a low-molecular-weight fatty acid that is FDA-approved for the treatment of urea cycle disorders. Recent studies have identified its efficacy in inhibiting endoplasmic reticulum (ER) stress and exerting anti-oxidative effects [19]. Our previous research demonstrated that PBA protects vital organ functions, including cardiovascular and renal homeostasis [20]. In the present study, we further confirm that PBA confers a protective role in HAUHS.

Compromised fluid tolerance is a prominent pathological feature of HAUHS, where excessive resuscitation fluid administration escalates the risk of pulmonary and cerebral edema [21, 22]. In the present study, lactated Ringer’s (LR) solution, which is the most commonly used and accessible crystalloid, particularly in resource-constrained settings, served as the standard intervention. While LR alone has the limitation of requiring large volumes for effective resuscitation [23], its co-administration with PBA significantly reduces total LR needs. This reduction in resuscitation volume mitigates edema formation, a critical benefit given the heightened susceptibility to fluid overload in hypoxic high-altitude environments, as evidenced by PBA’s ability to decrease pulmonary and cerebral water content.

Beyond fluid-sparing effects, this synergistic interaction holds particular value in prehospital settings where blood components are scarce or delayed. Notably, while HAUHS is associated with inherently lower blood loss compared to low-altitude counterparts for equivalent severity, PBA treatment further reduced blood loss during hypotensive resuscitation by approximately 13.3% relative to LR monotherapy. This dual benefit that preserving intravascular volume and diminishing bleeding reduces the need for urgent transfusion, is an essential advantage in resource-limited contexts. Clinically, such reduction is critical for maintaining hemodynamic stability: it prevents excessive loss of plasma proteins, a phenomenon that can trigger hemodilution, coagulopathy, and progression to the lethal triad (hypothermia, acidosis, coagulopathy). Collectively, these observations underscore PBA’s beneficial role in managing HAUHS, enhancing LR efficacy while addressing key challenges of fluid intolerance and limited resources. Consistent with the findings from Parts 2 and 3, our data confirm that early PBA administration protects against myocardial injury, hepatic damage, and renal dysfunction in rats with HAUHS—an effect previously validated in our prior work [20]. Mechanistically, PBA-mediated vital organ protection is linked to anti-endoplasmic reticulum stress and anti-oxidative stress pathways [20], while the current study further indicates this protection may stem from its ability to reduce blood loss and sustain superior hemodynamics, as evidenced by enhanced hepatic and renal blood flow (Fig. 3D, 3E).

Beyond these mechanistic insights, PBA’s preservation of vital organ function (heart, liver, kidney) during the prehospital phase holds profound clinical implications for subsequent definitive care. By attenuating cardiac injury (reduced cTnI), maintaining hepatic and renal perfusion, and improving oxygen delivery (DO2) and consumption (VO2), PBA primes organs to tolerate the physiological stress of delayed definitive treatment. This enhanced organ resilience is critical: it reduces the risk of post-resuscitation complications such as multi-organ dysfunction syndrome, which significantly increases mortality in UHS. Thus, PBA’s ability to improve organ perfusion and preserve function creates a favorable foundation for subsequent interventions, bridging the gap between prehospital stabilization and definitive care in high-altitude settings.

Our results indicate that PBA improves hemodynamic parameters in HAUHS more effectively than LR alone. Specifically, PBA stabilizes mean arterial pressure (MAP) within the 50–60 mmHg range during hypotensive resuscitation while reducing blood loss—critical factors for ensuring adequate blood volume, enhancing tissue perfusion, and mitigating ischemic injury to vital organs, particularly cardiac systolic function [24]. Notably, PBA treatment significantly reduced levels of the cardiac injury marker cTnI. Preserved cardiac function, in turn, improves systemic circulation and ensures perfusion of vital organs, as evidenced by the marked increase in hepatic and renal blood flow following PBA administration. Thus, PBA-mediated protection of cardiac function may represent a primary contributor to improved hemodynamics, though further studies are required to elucidate the specific mechanisms underlying this cardiac protection.

Notably, PBA extends the duration of effective hypotensive resuscitation (Fig S1A), thereby prolonging the prehospital window for safe evacuation to definitive care. In high-altitude scenarios where evacuation times are inherently prolonged, this extension could translate to higher survival rates by bridging the gap between injury and surgical hemostasis.

## Conclusion

In summary, PBA decreases the volume of resuscitation fluid requirements and extends the golden period (prehospital treatment window) by alleviating vital organ injury and reducing the risk of pulmonary and cerebral edema in resource-limited, high-altitude environments of the prehospital phase. These findings hold significant implications for managing UHS in high-altitude scenarios, including military operations, disaster relief, and traffic accidents.

## Supplementary Information

Supplementary material 1. Figure S1 A: the mean arterial pressure (MAP) (D) of rats with uncontrolled hemorrhagic shock during the effective hypotensive resuscitation stage (n=16). B: Venn diagram of overlapping targets between shock-related genes and PBA potential targets. C: Gene Ontology (GO) functional enrichment analysis of overlapping targets; D, E: Effects of PBA on plasma IL-6 and TNF-α concentrations in a rat model of high-altitude hemorrhagic shock (n=4). Data are means±SD. LR: Lactate Ringer’s solution.

## Data Availability

The datasets used during the current study are available from the corresponding author on reasonable request.
