# Supplementary figures and images for: 4-Phenylbutyric acid extends the gold time of uncontrolled hemorrhagic shock at high altitude by alleviating vital organ injury
Source: Intensive Care Med Exp. 2025 Dec 19;13:132. doi: 10.1186/s40635-025-00833-w (PMC12714675; doi:10.1186/s40635-025-00833-w)

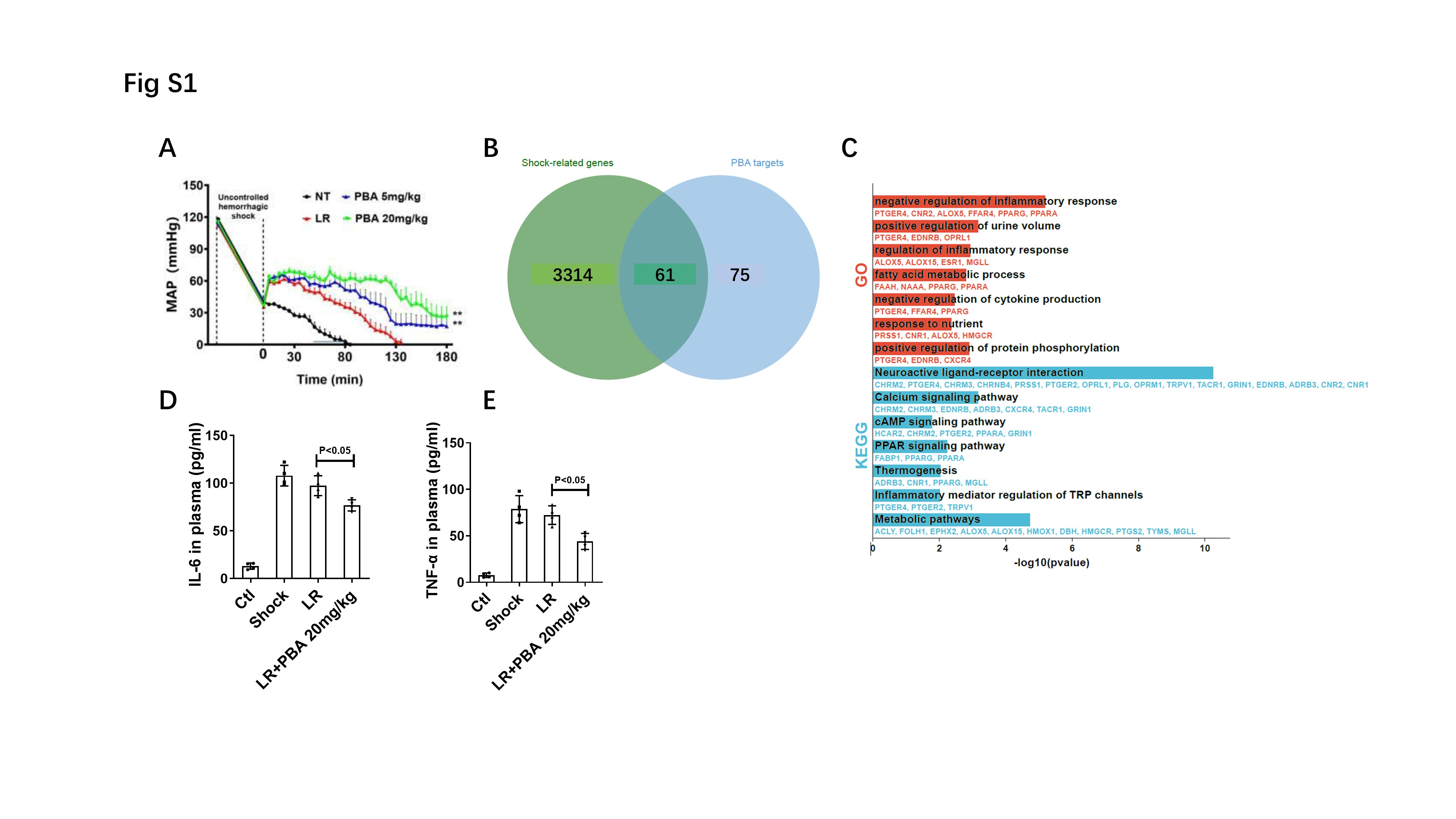

Supplement: Supplementary file 1 — Supplementary material 1. Figure S1 A: the mean arterial pressure (MAP) (D) of rats with uncontrolled hemorrhagic shock during the effective hypotensive resuscitation stage (n=16). B: Venn diagram of overlapping targets between shock-related genes and PBA potential targets. C: Gene Ontology (GO) functional enrichment analysis of overlapping targets; D, E: Effects of PBA on plasma IL-6 and TNF-α concentrations in a rat model of high-altitude hemorrhagic shock (n=4). Data are means±SD. LR: Lactate Ringer’s solution. [file 40635_2025_833_MOESM1_ESM.jpg]
